# Highly selective fluorescent and colorimetric probe for live-cell monitoring of sulphide based on bioorthogonal reaction

**DOI:** 10.1038/srep08969

**Published:** 2015-03-11

**Authors:** Fang-Jun Huo, Jin Kang, Caixia Yin, Jianbin Chao, Yongbin Zhang

**Affiliations:** 1 Research Institute of Applied Chemistry, Shanxi University, Taiyuan. 030006, China; 2 Key Laboratory of Chemical Biology and Molecular Engineering of Ministry of Education, Institute of Molecular Science, Shanxi University, Taiyuan 030006, China

## Abstract

H_2_S is the third endogenously generated gaseous signaling compound and has also been known to involve a variety of physiological processes. To better understand its physiological and pathological functions, efficient methods for monitoring of H_2_S are desired. Azide fluorogenic probes are popular because they can take place bioorthogonal reactions. In this work, by employing a fluorescein derivative as the fluorophore and an azide group as the recognition unit, we reported a new probe 5-azidofluorescein for H_2_S with improved sensitivity and selectivety. The probe shows very low background fluorescence in the absence of H_2_S. In the presence of H_2_S, however, a significant enhancement for excited fluorescence were observed, resulting in a high sensitivity to H_2_S in buffered (10 mmol/L HEPES, pH 7.0) aqueous acetonitrile solution (H_2_O/CH_3_CN = 1:3, v/v) with a detection limit of 0.035 *μ*mol/L observed, much lower than the previously reported probes. All these features are favorable for direct monitoring of H_2_S with satisfactory sensitivity, demonstrating its value of practical application.

Fluorogenic probes activated by bioorthogonal chemical reactions can enable biomolecule imaging in situations where it is not possible to wash away unbound probe^1^. Much work has been devoted to expanding the toolbox of bioorthogonal reactions, and these efforts can be complemented by the development of fluorogenic probes^2^. Such probes are typically endowed with a functionality that suppresses fluorescence. Its transformation during the reaction creates a new functionality that no longer quenches the fluorescence of the underlying system, resulting in a fluorescence enhancement. Such probes offer significant advantages for imaging studies in which it is not possible to wash away unreacted probe, such as real-time imaging of dynamic processes in cells or visualization of molecules in live organisms.

One of the most widely used bioorthogonal reactions is the azide−alkyne [3 + 2] cycloaddition to form a triazole^3^,^4^. This reaction has enabled the selective visualization of azide- or alkyne-labeled proteins, glycans, nucleic acids, and lipids^4^,^5^. Several azide-6,7,8,9 fluorogenic probes have been reported, largely based on coumarins^6^,^10^, naphthalimides^8^, and other systems that require UV excitation and emit blue light^7^,^11^,^12^. Such wavelengths are not ideal for biological imaging because of high levels of autofluorescence and poor tissue penetrance^13^.

An obvious improvement upon these designs would be the development of azido fluorogenic probes with longer excitation and emission wavelengths. Some attempts at achieving this goal have been made^8^,^12^,^14^. The utility of azide pairs in biological settings remains unclear. Thus, fluorogenic azido probes that perform well as cell-imaging reagents remain an important goal. Bertozzi reported the rational design and experimental validation of azide-functionalized fluorogenic probes based on the widely used blue-excitation/green-emission fluorescein scaffold^15^. In their work, they have prepared a series of azidefluorescein compounds under NaNO_2_/NaN_3_ condition, and the azidefluorescein was used to biological imaging in Chinese hamster ovary (CHO) cells labled with alkynylsialic acid nor H_2_S.

It is well known that H_2_S have been demonstrated to exert protective effects in many pathologies and physiologies^16^,^17^,^18^,^19^,^20^,^21^,^22^,^23^,^24^,^25^,^26^. So the discovery of these emerging biological roles of H_2_S has resulted in rising interest in H_2_S research. Accordingly, rapid, accurate and reliable methods for H_2_S detection are in high demand, as they have potential to provide useful information for better understanding its biological functions^27^. And simple, specific, and real-time analytic methods/sensors are highly desirable for H_2_S in biological systems. In fact, it is a good choice to introduce an azido group into probes to be reduced by H_2_S due to the simple synthesis, relatively good selectivity, suitable reaction time, and non-cell toxicity^28^,^29^,^30^,^31^,^32^,^33^,^34^.

With these considerations in mind, we also prepared 5-azidefluorescein from 5-aminofluorescein under NaNO_2_/NaN_3_ condition according to literature^15^ (Fig. 1) and tried to use this compound to detect H_2_S. It is delightful that we obtained the crystal of 5-aminofluorescein and found probe can be used as a high selective and sensitive fluorescent probe for H_2_S firstly. Furthermore, the probe also was applied in cell imaging.

5-aminofluorescein (0.35 g, 1 mmol), a deep-red solid, was dissolved in 10 mL 2:1 AcOH/H_2_O and cooled to 0°C. To this deep red solution was added NaNO_2_, a white powder (0.10 g, 1.5 mmol). After stirring for 15 minutes, the solution had turned to a light red color. NaN_3_ (0.10 g, 1.5 mmol) was then carefully added (caution: gas evolution!), turning the solution to a yellow slurry. The reaction was stirred for 2 hr at 0°C. The slurry was filtered over vacuum and the solid washed with 20 mL 2 mol/L HCl and 100 mL H_2_O, yielding 5-azidofluoresceinquinone (0.30 g, 80%) as a yellow solid after further drying in vacuo and characterized by NMR, ESI-MS, elemental analysis, X-ray crystal diffractometer (see Figure S1).

Reaction of probe (1 *μ*mol/L) with Na_2_S (2 *μ*mol/L) as an aqueous sulphide source at room temperature in buffered (10 mmol/L HEPES, pH 7.0) aqueous acetonitrile solution (H_2_O/CH_3_CN = 1:3, v/v) yielded a time-dependent fluorescence increase, which was completed within 5 s (Supplementary Fig. S2). Δ*F* > 50-fold increase in the fluorescence intensity accompanied (*Φ* = 0.35) with a green emission at 531 nm. However, the analytes without hydrogen sulfide induced no changes in the fluorescence emission properties under the same conditions (Fig. 2a). The competing experiments indicated other analytes did not disturb the determination for sulphide (Fig. 2b). It is noted that the unprecedented speed of this probe's response and high selectivity compared with other probes^35^,^36^,^37^,^38^ suggests the possibility of quantitative detection without the need for sample pretreatment. The results reason that H_2_S-mediated reduction of azides to amines would generate highly fluorescent products (Fig. 3)^39^. H_2_S-induced product was confirmed its molecular formula by electrospray ionization mass spectrometry (ESI-MS). The peak at m/z 346.42 corresponding to [5-aminofluorescein-H]^+^, was clearly observed (Supplementary Fig. S3). Further ^1^HNMR spectroscopic analysis also provided the evidence for the product of 5-aminofluorescein. With addition of 2 equiv. of Na_2_S (containing crystal water) to probe in DMSO-*d*_6_ (Fig. S4), the resonance of the original proton (azidebenzene CH) at 7.28 and 7.49 ppm all shifted to upfield owing to presence of electron- pushing group NH_2_ (Supplementary Fig. S4) and appeared at 6.65 ~ 6.76 ppm.

Next, varying concentrations of Na_2_S (0–2.0 *μ*mol/L) were added to the test reaction solution. The fluorescence intensity increased linearly with the concentration of Na_2_S up to 2.0 *μ*mol/L, and, thereafter, reached a steady state (Fig. 4). The detection limit, based on the definition by IUPAC (C_DL_ = 3 S_b_/m)^40^, was found to be 0.035 *μ*mol/L from 10 blank solutions (Supplementary Fig. S5). This probe therefore shows a high sensitivity toward sodium sulfide comparable to that of other reported S_2_−chemosensors^35^,^36^,^37^,^38^ (Table 1).

We also performed absorption spectral experiments in the buffered (10 mmol/L HEPES, pH 7.0) aqueous acetonitrile solution (H_2_O/CH_3_CN = 1:3, v/v) containing probe (8 *μ*mol/L) when the H_2_S was added gradually. Fig. S6 showed absorbance changes of probe in the buffered (10 mmol/L HEPES, pH 7.0) aqueous acetonitrile solution (H_2_O/CH_3_CN = 1:3, v/v) after the addition of 4 equiv. of H_2_S. The probe has no absorbance at UV-Vis area, immediately there generated an absorbance at 510 nm and the absorbance intensity enhanced with increased H_2_S corresponding solution color change from colorless to yellow. The notable variation was ended after about 4 equiv. of H_2_S added, relating to the H_2_S-mediated reduction of 5-azidefluoresceinquinone to 5-aminofluorescein (ring-open).

Most publications suggest that the average endogenous H_2_S level is in the *μ*mol/L range^31^,^32^,^41^, Since the detection limit of this probe was found to be 0.035 *μ*mol/L, thus it become possible that the probe can detect H_2_S level in tissue imaging. The ability of probe to detect sulphide within living cells was also evaluated by laser confocal fluorescence imaging using a Leica TCS SP5 laser scanning microscope. Imaging of sulphide substrates in HeLa cells after 30 min incubation using probe (2 μmol/L) showed weak green fluorescence (Fig. 5b). HepG2 cells incubated with 2 μmol/L probe for 30 min at 37°C, and with 4 *μ*mol/L exogenous H_2_S for another 30 min at 37°C, showed green fluorescence (Fig. 5c) (it is noted that 30 min was usually selected in cell imaging experiment). We also carried out time course experiment in the cell. Fig. 6 (left) indicated that a 15 min is enough for cell permeability (Fig. 6h) reaction and the cell can survive even if in a 45 min after H_2_S was added (Fig. 6i). In addition, according to the Qian's method^42^, we employed sodium nitroprusside (SNP, a NO donor) to stimulate the production of endogenous H_2_S in cells^43^. With the addition of probe into the culture of the SNP (100 *μ*mol/L or 200 *μ*mol/L)-loaded cells for 20 min, a drastic increase of emission intensity (Fig. 6l, 6m), indicating the generation of endogenous H_2_S within the cells. These results demonstrate that this probe is selective for sulphide and amenable for live-cell imaging.

The development of innovative fluorescent imaging probes has revolutionized cell biology, allowing localization and dynamic monitoring of cellular metabolite and inorganic ion pools^43^,^44^,^45^. Recently, fluorescence and/or colorimetric chemosensors for H_2_S/aqueous sulphide based on some reaction mechanisms between probes and H_2_S have been reported. These include the cleavage of alcoxyl(R-O) bond^45^,^46^,^47^, the cleavage of S-O bond^47^,^48^,^49^, copper displacement approach^49^,^50^,^51^, nucleophilic addition approach^5^,^38^,^52^,^53^. A significant bottleneck in the above emerging field of H_2_S/aqueous sulphide signalling is the absence of technology for effective in vivo detection and imaging. And this problem is exacerbated by fact that similar substances such as sulphide which contain SH group may mislead the detection of intracellular thiol concentration. In this study, we have successfully developed an azide-to-amine reduction chemical strategy for selective sulphide detection, which can be used to monitor sulphide generation in live cells in the presence of large excess of thiols. We show that the same chemistry can be readily adapted to different fluorescent templates for sulphide detection and imaging. The same chemistry will lead to new probes with faster response, which may help to monitor fluctuations of H_2_S in situ. Further optimization and utilization of this strategy and this class of probes should dramatically accelerate future studies of H_2_S in biology.

## Methods

4-(2-Hydroxyethyl)-1-piperazineethanesulfonic acid (HEPES) and sodium nitroprusside were purchased from Sigma–Aldrich (St. Louis, MO). Sodium hydroxide solution (0.1 mol/L) was added to aqueous HEPES (10 mmol/L) to adjust the pH to 7.0. Anionic salts were purchased from Shanghai Experiment Reagent Co., Ltd (Shanghai, China). All other chemicals used were of analytical grade. Deionized water was used to prepare all aqueous solutions. The solutions of anions were prepared from their sodium salts.

### Instruments

A pH meter (Mettler Toledo, Switzerland) was used to determine the pH. Fluorescence spectra were measured on Cary Eclipse fluorescence spectrophotometer. A PO-120 quartz cuvette (10 mm) was purchased from Shanhai Huamei Experiment Instrrument Plants, China. ESI-MS was measured with an UPLC-ESI-Q-TOF synapt G2 (Waters) instrument. The ability of probe reacting to hydrogen sulfide in the living cells was also evaluated by laser confocal fluorescence imaging using a Leica TCS SP5 laser scanning microscope.

### Imaging of HepG2 cells

The HepG2 cells were grown in 1 × SPP medium (1% proteose peptone, 0.2% glucose, 0.1% yeast extract, 0.003% EDTA ferric sodium salt) at 37°C. The HepG2 were treated with 2 *μ*mol/L of probe (methanol stock solution) in culture media for 30 min at 37°C and washed 3 times with PBS. The HepG2 cells were first incubated with 2 *μ*mol/L of probe for 30 min at 37°C and with 20 *μ*M exogenous H_2_S for final 30 min at 37°C.

## Author Contributions

F.J. and C.X. conceived the idea and directed the work. Y.B. designed experiments. J.K. performed the synthesis and in vitro tests. J.B. carried out NMR experiment. All authors contributed to data analysis and manuscript writing.

## Supplementary Material

Supplementary Information Supplementary Information

## Figures and Tables

**Figure 1:**
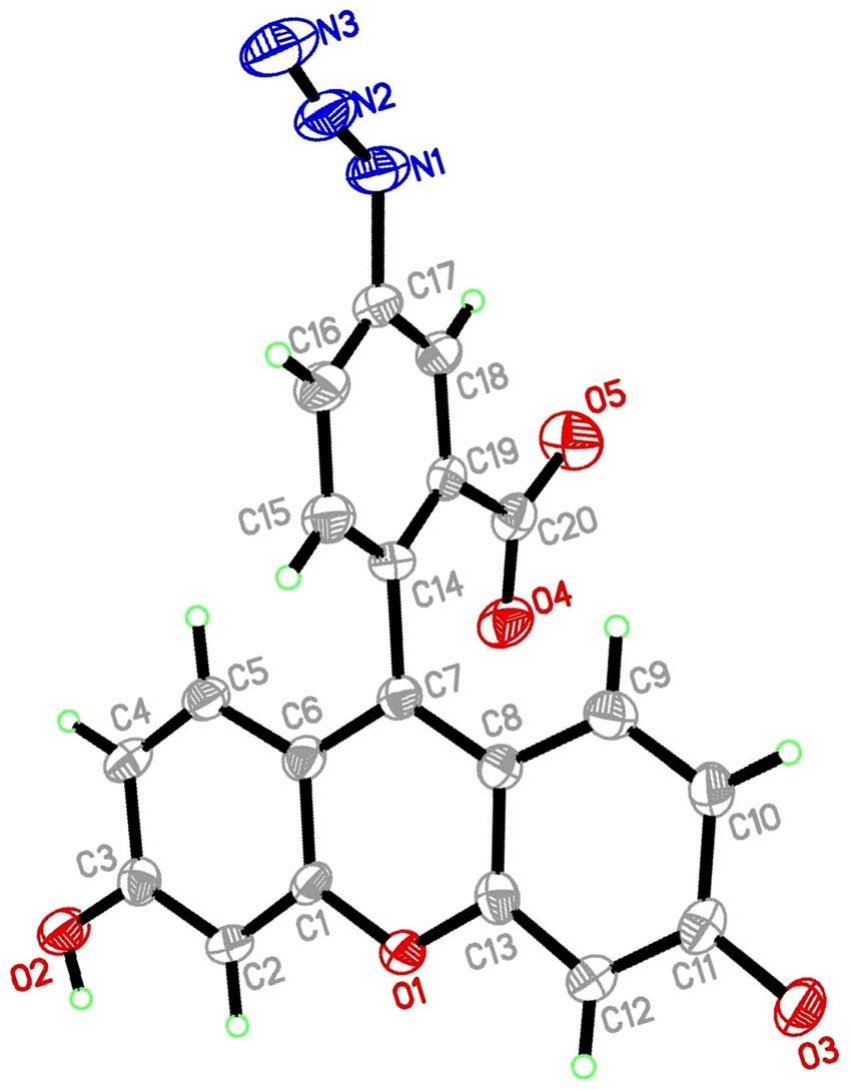
Structure and thermal ellipsoids of probe are drawn at the 50% probability level.

**Figure 2:**
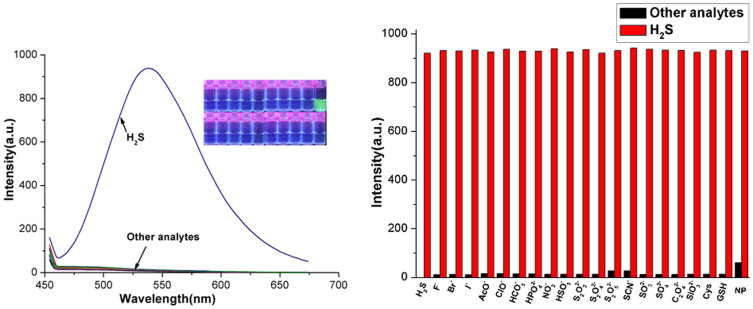
(a) Fluorescence spectra of probe (1 *μ*mol/L) with various analytes (20 *μ*mol/L) in water:CH_3_CN (1:3 v/v, HEPES buffer, pH 7.0) solutions (*λ*_ex_ = 425 nm, slit: 5 nm/5 nm), inset: a visual fluorescence change photograph for H_2_S (green) and other analytes (colorless) under illumination with a 365 nm UV lamp; (b) Relative fluorescent intensity (*λ*_ex_ = 425 nm, *λ*_em_ = 531 nm) of the system. (black bar: various analytess, red bar: probe + various analytes + H_2_S).

**Figure 3:**
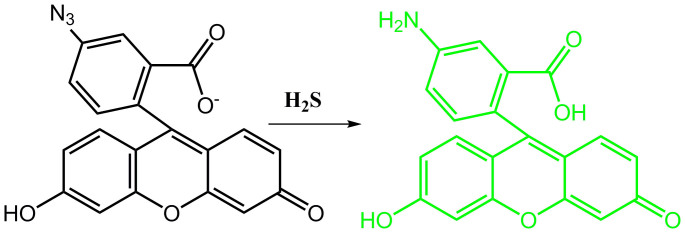
The proposed mechanism for the determination of H_2_S.

**Figure 4:**
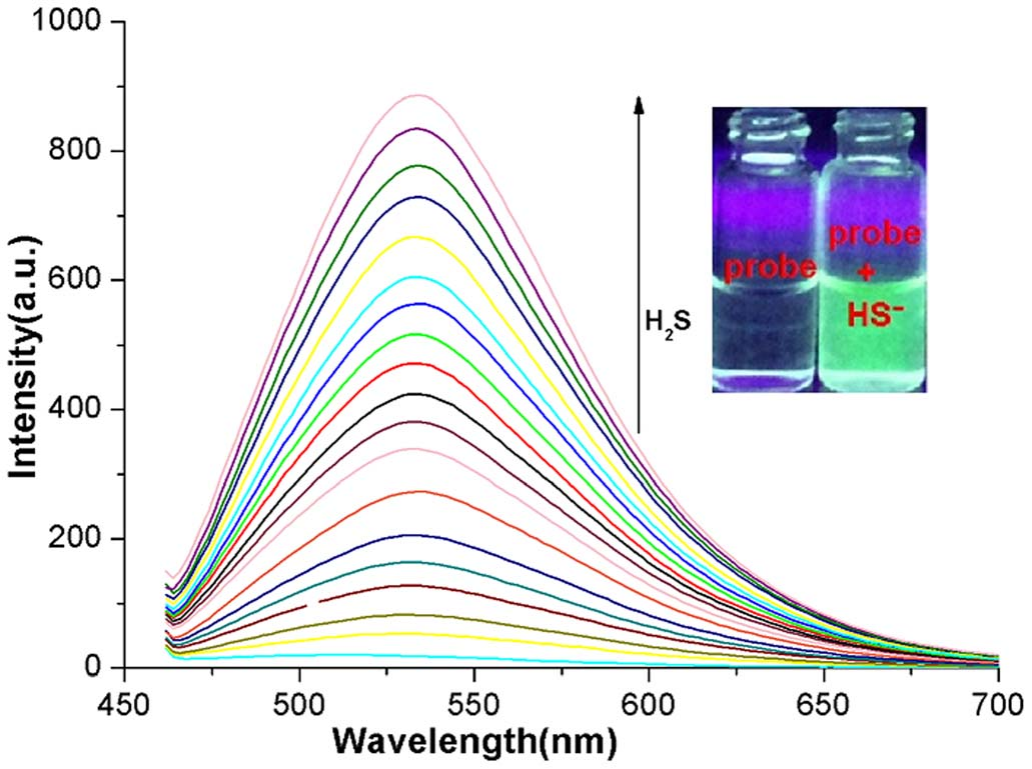
Fluorescence spectra of probe (1 *μ*mol/L) in the presence of various concentrations of H_2_S (0-2.0 *μ*mol/L) in water:CH_3_CN (1:3 v/v, HEPES buffer, pH 7.0) solution. (*λ*_ex_ = 425 nm, slit: 5 nm/5 nm); each spectrum is recorded 0.5 min after H_2_S addition.

**Figure 5:**
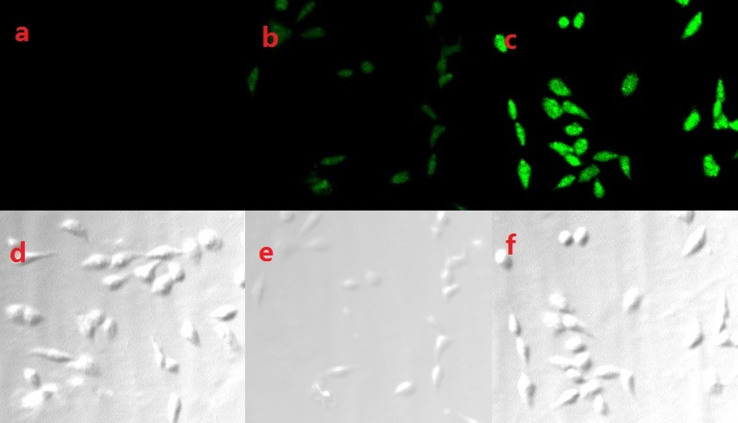
Confocal fluorescence images in HepG2 cells. (a) absence and presence of 2 *μ*mol/L probe (b); (c) Fluorescence image of HepG2 cells incubated with 2 *μ*mol/L probe for 30 min at 37°C and then incubated with 4 *μ*mol/L H_2_S for 30 min at 37°C; (d), (e), (f) were their brightfield images of (a), (b), (c).

**Figure 6:**
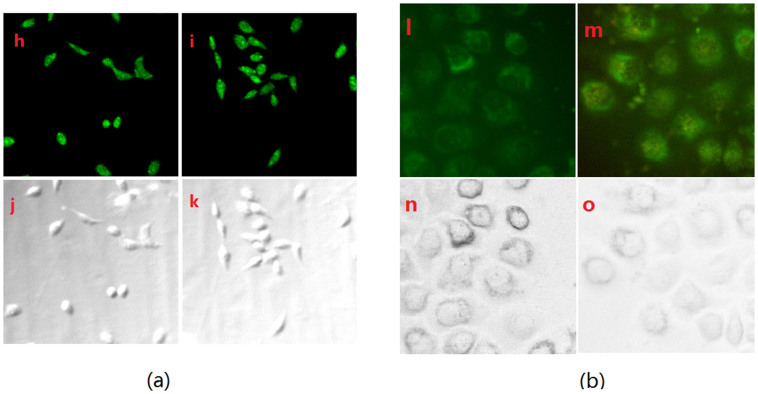
(a) The time dependence of confocal fluorescence images of exogenous sulphide in HepG2 cells. (h) and (i) Fluorescence image of HepG2 cells incubated with 2 *μ*mol/L probe for 30 min at 37°C and then incubated with 4 *μ*mol/L H_2_S for 15 min and 45 min at 37°C, respectively; (j), (k) were their brightfield images of (h), (i); (b) Confocal fluorescence images of endogenous H_2_S in living HepG2 cells with probe (2 *μ*mol/L) upon excitation at 425 nm. Cells were prestimulated with SNP (100 *μ*M) for 30 min, then incubated with probe (2 *μ*mol/L) for 20 min (l, n). Cells were prestimulated with SNP (200 *μ*mol/L) for 30 min, and then incubated with probe (2 *μ*mol/L) for 20 min (m, o).

**Table 1:** A compared table about the detection limits and time course for H_2_S

Method	Analyte	Signal output	Solvent	Detection Limit (*μ*mol/L)	Response Time	Time course in cell
Ref. 35	H_2_S	Fluorescence	HEPES buffer	0.08	20 min	50 min
Ref. 38	H_2_S	Fluorescence	PBS-DMSO(1:1, v/v, pH 7.4)	3.05	40 min	120 min
Ref. 36	H_2_S	Fluorescence	PBS–CH_3_CN (1:1, v/v, pH 7.4)	2.5	10 min	30 min
Ref. 37	H_2_S	Fluorescence	PIPES buffer (pH 7.4)	2.4	30 min	60 min
This work	H_2_S	Fluorescence	HEPES:CH_3_CN (1:3 v/v, pH 7.0)	0.035	10 s	30 min

## References

[b1] Sletten E. M. & Bertozzi C. R. Bioorthogonal chemistry: fishing for selectivity in a sea of functionality. Angew. Chem. Int. Ed. 48, 6974–6978 (2011). 10.1002/anie.200900942 PMC2864149 19714693

[b2] Le Droumaguet C. *et al.* Fluorogenic click reaction. Chem. Soc. Rev. 39, 1233–1239 (2010). 20309483 10.1039/b901975h

[b3] Best M. D. Click Chemistry and Bioorthogonal Reactions: Unprecedented Selectivity in the Labeling of Biological Molecules. Biochem. 48, 6571–6584 (2009). 19485420 10.1021/bi9007726

[b4] Jewett J. C. & Bertozzi C. R. Cu-free click cycloaddition reactions in chemical biology. Chem. Soc. Rev. 39, 1272–1279 (2010). 20349533 10.1039/b901970g PMC2865253

[b5] Best M. D. Click Chemistry and Bioorthogonal Reactions: Unprecedented Selectivity in the Labeling of Biological Molecules. Biochem. 48, 6571–6584 (2009). 19485420 10.1021/bi9007726

[b6] Sivakumar K. *et al.* Fluorogenic 1, 3-Dipolar Cycloaddition Reaction of 3-Azidocoumarins and Acetylenes. Org. Lett. 6, 4603–4606 (2004). 15548086 10.1021/ol047955x

[b7] Xie F. *et al.* A fluorogenic ‘click' reaction of azidoanthracene derivatives. Tetrahedron 64, 2906–2914 (2008).

[b8] Wang C. *et al.* Tuning the optical properties of BODIPY dye through Cu (I) catalyzed azide-alkyne cycloaddition (CuAAC) reaction. Sci. China Chem. 55, 125–130 (2012).

[b9] Sawa M. *et al.* Glycoproteomic probes for fluorescent imaging of fucosylated glycans in vivoProc. Natl. Acad. Sci. U.S.A. 103, 12371–12376 (2006). 10.1073/pnas.0605418103 PMC1567886 16895981

[b10] Zhou Z. & Fahrni C. J. A. Fluorogenic Probe for the Copper (I)-Catalyzed Azide−Alkyne Ligation Reaction: Modulation of the Fluorescence Emission via 3(n,π*)−1(π,π*) Inversion. J. Am. Chem. Soc. 126, 8862–8863 (2004). 15264794 10.1021/ja049684r

[b11] Key J. A. & Cairo C. W. Identification of fluorogenic and quenched benzoxadiazole reactive chromophores. Dyes Pigm. 88, 95–102 (2010).

[b12] Qi J. *et al.* Developing Visible Fluorogenic ‘Click-On' Dyes for Cellular Imaging. Bioconjugate Chem. 22, 1758–1762 (2011). 10.1021/bc200282t PMC3178751 21823670

[b13] Weissleder R. & Ntziachristos V. Shedding light onto live molecular targets. Nat. Med. 9, 123–128 (2003). 12514725 10.1038/nm0103-123

[b14] Li J. *et al.* Rapid Synthesis, Screening, and Identification of Xanthone- and Xanthene-Based Fluorophores Using Click Chemistry. Org. Lett. 11, 3008–3011 (2009). 19522535 10.1021/ol9010344

[b15] Shieh P. *et al.* Fluorogenic Azidofluoresceins for Biological Imaging. J. Am. Chem. Soc. 134, 17428–17431 (2012). 23025473 10.1021/ja308203h PMC3596100

[b16] Qian Y. *et al.* Selective fluorescent probes for live-cell monitoring of sulphide. Nat. Commun. 2, 1506–1512 (2011). 10.1038/ncomms1506 21988911

[b17] Chen W. *et al.* New fluorescent probes for sulfane sulfurs and the application in bioimaging. Chem. Sci. 4, 2892–2896 (2013). 23750317 10.1039/C3SC50754H PMC3673728

[b18] Papapetropoulos A. *et al.* Hydrogen sulfide is an endogenous stimulator of angiogenesis. Proc. Natl. Acad. Sci. U.S.A. 106, 21972–21977 (2009). 19955410 10.1073/pnas.0908047106 PMC2799889

[b19] Yang G. *et al.* H_2_S as a Physiologic Vasorelaxant: Hypertension in Mice with Deletion of Cystathionine γ-Lyase. Science 322, 587–590 (2008). 18948540 10.1126/science.1162667 PMC2749494

[b20] Yang G. *et al.* Pro-apoptotic effect of endogenous H2S on human aorta smooth muscle cells. FASEB J. 20, 553–555 (2006). 16507767 10.1096/fj.05-4712fje

[b21] Li L. *et al.* Hydrogen sulfide is a novel mediator of lipopolysaccharide-induced inflammation in the mouse. FASEB J. 19, 1196–1198 (2005). 15863703 10.1096/fj.04-3583fje

[b22] Abe K. & Kimura H. The possible role of hydrogen sulfide as an endogenous neuromodulator. J. Neurosci. 16, 1066–1071 (1996). 8558235 10.1523/JNEUROSCI.16-03-01066.1996 PMC6578817

[b23] Eto K. *et al.* Brain hydrogen sulfide is severely decreased in alzheimer's disease. Biochem. Biophys. Res. Commun. 293, 1485–1488 (2002). 12054683 10.1016/S0006-291X(02)00422-9

[b24] Kamoun P. *et al.* Endogenous hydrogen sulfide overproduction in Down syndrome. Am. J. Med. Genet. 116A, 310–311 (2003). 12503113 10.1002/ajmg.a.10847

[b25] Yang W. *et al.* Activation of KATP channels by H_2_S in rat insulin-secreting cells and the underlying mechanisms. J. Physiol. 569, 519–531 (2005). 16179362 10.1113/jphysiol.2005.097642 PMC1464240

[b26] Fiorucci S. *et al.* The third gas: H_2_S regulates perfusion pressure in both the isolated and perfused normal rat liver and in cirrhosis. J. Hepatol. 42, 539–548 (2005). 10.1002/hep.20817 16108046

[b27] Liu Y. & Feng G. A visible light excitable colorimetric and fluorescent ESIPT probe for rapid and selective detection of hydrogen sulfide. Org. Biomol. Chem. 12, 438–445 (2014). 24263381 10.1039/c3ob42052c

[b28] Peng H. *et al.* A Fluorescent Probe for Fast and Quantitative Detection of Hydrogen Sulfide in Blood. Angew. Chem. Int. Ed. 50, 9672–9675 (2011). 10.1002/anie.201104236 PMC3529136 21882324

[b29] Montoya L. A. & Pluth M. D. Selective turn-on fluorescent probes for imaging hydrogen sulfide in living cells. Chem. Commun. 48, 4767–4769 (2012). 10.1039/c2cc30730h PMC3340910 22473176

[b30] Das S. *et al.* A small molecule two-photon probe for hydrogen sulfide in live tissues. Chem. Commun. 48, 8395–8397 (2012). 10.1039/c2cc33909a 22797290

[b31] Chen S. *et al.* Reaction-Based Genetically Encoded Fluorescent Hydrogen Sulfide Sensors. J. Am. Chem. Soc. 134, 9589–9592 (2012). 22642566 10.1021/ja303261d

[b32] Lippert A. R. *et al.* Reaction-Based Fluorescent Probes for Selective Imaging of Hydrogen Sulfide in Living Cells. J. Am. Chem. Soc. 133, 10078–11080 (2011). 21671682 10.1021/ja203661j

[b33] Yu F. *et al.* An ICT-based strategy to a colorimetric and ratiometric fluorescence probe for hydrogen sulfide in living cells. Chem. Commun. 48, 2852–2854 (2012). 10.1039/c2cc17658k 22293939

[b34] Wan Q. *et al.* In vivo monitoring of hydrogen sulfide using a cresyl violet-based ratiometric fluorescence probe. Chem. Commun. 49, 502–504 (2013). 10.1039/c2cc37725j 23202298

[b35] Yu F. *et al.* An ICT-based strategy to a colorimetric and ratiometric fluorescence probe for hydrogen sulfide in living cells. Chem. Commun. 48, 2852–2854 (2012). 10.1039/c2cc17658k 22293939

[b36] Sun K., Liu X., Wang Y. & Wu Z. A polymer-based turn-on fluorescent sensor for specific detection of hydrogen sulfide. RSC Adv. 3, 14543–14548 (2013).

[b37] Jiang Y., Wu Q. & Chang X. A ratiometric fluorescent probe for hydrogen sulfide imaging in living cells. Talanta 121, 122–126 (2014). 24607118 10.1016/j.talanta.2014.01.001

[b38] Sun W. *et al.* A two-photon fluorescent probe with near-infrared emission for hydrogen sulfide imaging in biosystems. Chem. Commun. 49, 3890–3892 (2013). 10.1039/c3cc41244j 23549590

[b39] Bailey T. S. & Pluth M. D. Chemiluminescent Detection of Enzymatically Produced Hydrogen Sulfide: Substrate Hydrogen Bonding Influences Selectivity for H_2_S over Biological Thiols. J. Am. Chem. Soc. 135, 16697–16704 (2013). 24093945 10.1021/ja408909h PMC3868629

[b40] Ding Y. B. *et al.* α-Monoacylated and α,α′- and α,β′-Diacylated Dipyrrins as Highly Sensitive Fluorescence “Turn-on” Zn^2+^ Probes. J. Org. Chem. 78, 5328–5338 (2013). 23668855 10.1021/jo400454e

[b41] Xuan W. *et al.* Fluorescent Probes for the Detection of Hydrogen Sulfide in Biological Systems. Angew. Chem. Int. Ed. 51, 2282–2284 (2012). 10.1002/anie.201107025 22278769

[b42] Zhang L. *et al.* A colorimetric and ratiometric fluorescent probe for the imaging of endogenous hydrogen sulphide in living cells and sulphide determination in mouse hippocampus. Org. Biomol. Chem. 12, 5115–5125 (2014). 24874797 10.1039/c4ob00285g

[b43] Wolfe S. K., Andrade C. & Swinehart J. H. Kinetic studies of the pentacyanonitrosylferrate(2-) azide and hydroxylamine reactions. Inorg. Chem. 13, 2567–2572 (1974).

[b44] Yi L. *et al.* A highly sensitive fluorescence probe for fast thiol-quantification assay of glutathione reductase. Angew. Chem. Int. Ed. Engl. 48, 4034–4037 (2009). 19388016 10.1002/anie.200805693

[b45] Que E. L., Domaille D. W. & Chang C. J. Metals in neurobiology: probing their chemistry and biology with molecular imaging. Chem. Rev. 108, 1517–1549 (2008). 18426241 10.1021/cr078203u

[b46] Chen S., Chen Z.-J., Ren W. & Ai H.-W. Reaction-Based Genetically Encoded Fluorescent Hydrogen Sulfide Sensors. J. Am. Chem. Soc. 134, 9589 (2012). 22642566 10.1021/ja303261d

[b47] Cao X., Lin W., Zheng K. & He L. A near-infrared fluorescent turn-on probe for fluorescence imaging of hydrogen sulfide in living cells based on thiolysis of dinitrophenyl ether. Chem. Commun. 48, 10529–10531 (2012). 10.1039/c2cc34031c 22992474

[b48] Yang X. F., Wang L., Xu H. M. & Zhao M. L. A fluorescein-based fluorogenic and chromogenic chemodosimeter for the sensitive detection of sulfide anion in aqueous solution. Anal. Chim. Acta 631, 91–95 (2009). 19046684 10.1016/j.aca.2008.10.037

[b49] Fu L. *et al.* A ratiometric “two-in-one” fluorescent chemodosimeter for fluoride and hydrogen sulfide. Sens. Actuators B 193, 701–707 (2014).

[b50] Gu X. F., Liu C. H., Zhu Y. C. & Zhu Y. Z. Development of a boron-dipyrromethene-Cu^2+^ ensemble based colorimetric probe toward hydrogen sulfide in aqueous media. Tetrahedron Lett. 52, 5000–5003 (2011).

[b51] Zhang D. & Jin W. Highly selective and sensitive colorimetric probe for hydrogen sulfide by a copper (II) complex of azo-dye based on chemosensing ensemble approach. Spectrochimica Acta Part A 90, 35–39 (2012). 10.1016/j.saa.2012.01.013 22306448

[b52] Qian Y. *et al.* A fluorescent probe for rapid detection of hydrogen sulfide in blood plasma and brain tissues in mice. Chem. Sci. 3, 2920–2923 (2012).

[b53] Wang X. *et al.* A near-infrared ratiometric fluorescent probe for rapid and highly sensitive imaging of endogenous hydrogen sulfide in living cells. Chem. Sci. 4, 2551–2556 (2013).

