# Supplementary material for: Highly selective fluorescent and colorimetric probe for live-cell monitoring of sulphide based on bioorthogonal reaction
Source: Sci Rep. 2015 Mar 11;5:8969. doi: 10.1038/srep08969 (PMC4355735; doi:10.1038/srep08969)

**Supporting Information (SI)**

Highly selective fluorescent and colorimetric probe for live-cell monitoring of sulphide based on bioorthogonal reaction

§Fang-Jun Huo, ^1^ §Jin Kang, ^1^ Caixia Yin, ^2^,* Jianbin Chao^1^ & Yongbin Zhang ^1^

*^1^Research Institute of Applied Chemistry, Shanxi University, Taiyuan, 030006, China.*

*^2^ Key Laboratory of Chemical Biology and Molecular Engineering of Ministry of Education, Institute of Molecular Science, Shanxi University, Taiyuan 030006, China.*

*Corresponding author: C.X. Yin, E-mail: [yincx@sxu.edu.cn](mailto:yincx@sxu.edu.cn), Tel/Fax: +86-351-7011022;

§Fangjun Huo and Jin Kang contributed equally.

**Figure S1:** ^1^H NMR, ^13^C NMR, ESI-MS of probe

**Figure S2:** Plot of the fluorescence intensity (at 531 nm) as a function of the concentrations of H_2_S

**Figure S3:** ESI-MS spectra of the product after probe reacting with H_2_S

**Figure S4:** ^1^H NMR, ^13^C NMR spectra of the product after probe reacting with H_2_S

**Figure S5:** Detection limit for H_2_S from probe

**Figure S6:** UV-Vis absorption spectra upon H_2_S addition into probe

**Figure S1:** ^1^H NMR, ^13^C NMR, ESI-MS of probe


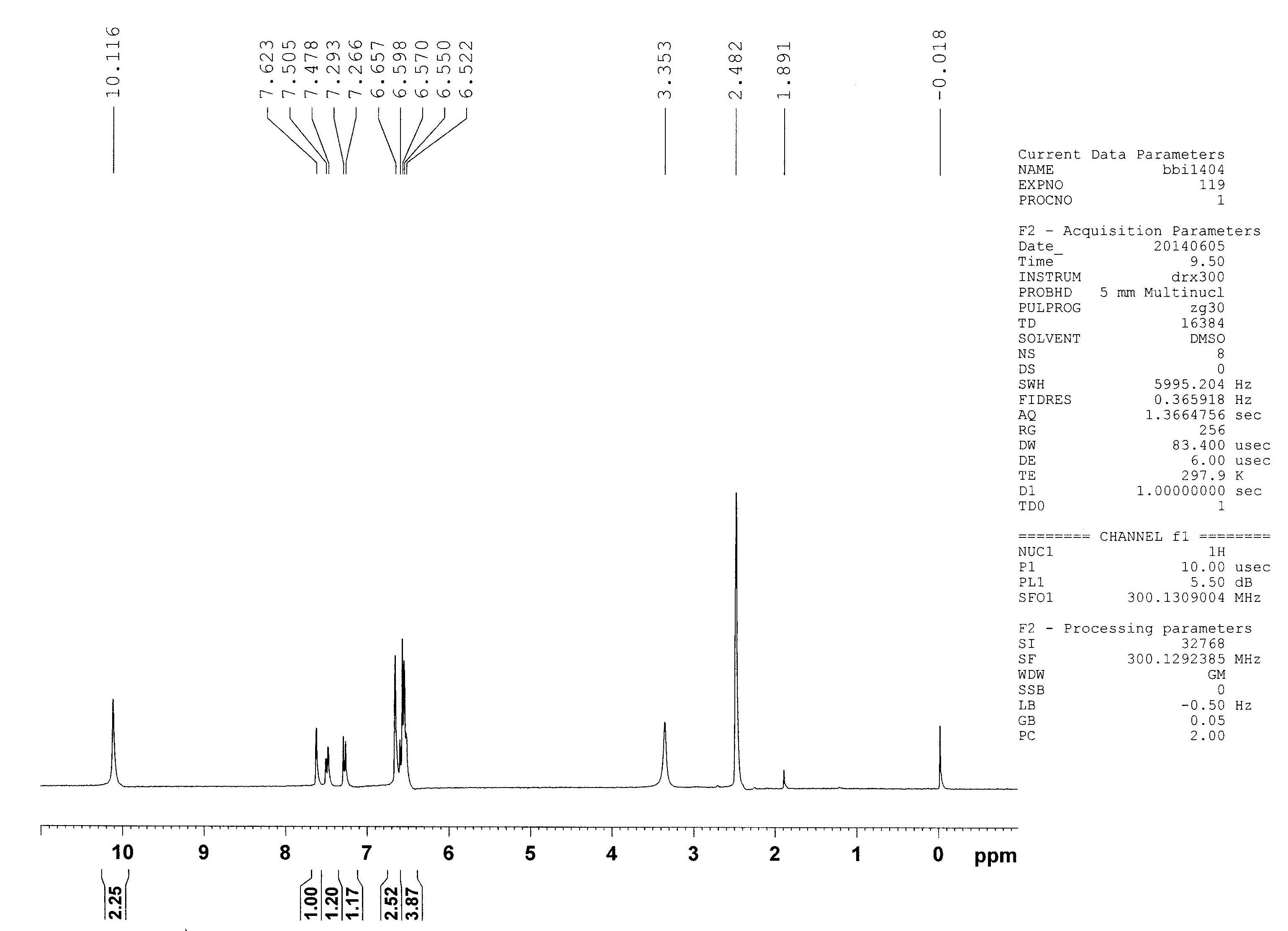


ESI-MS of the probe-HS^-^.


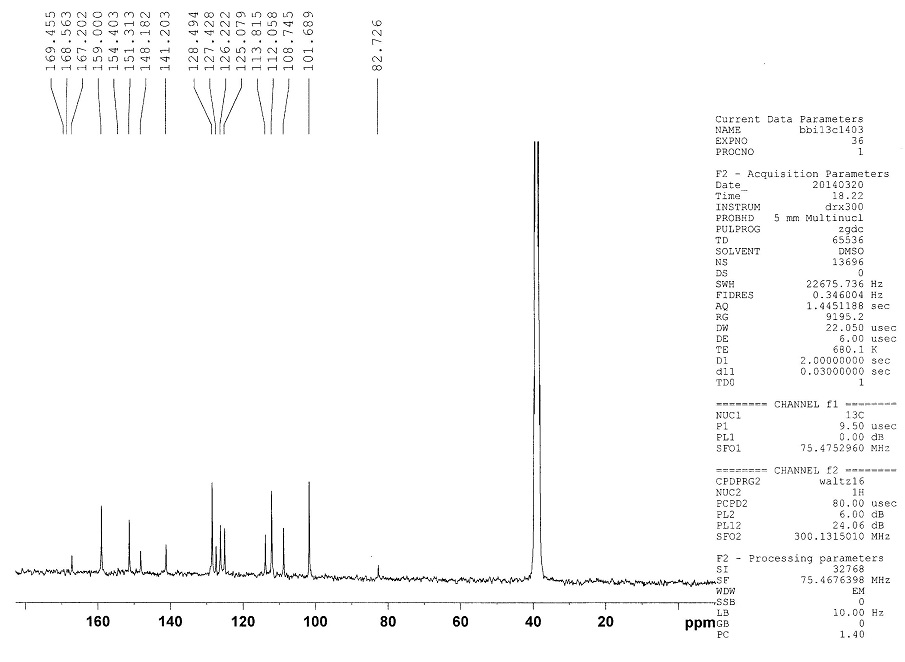


**
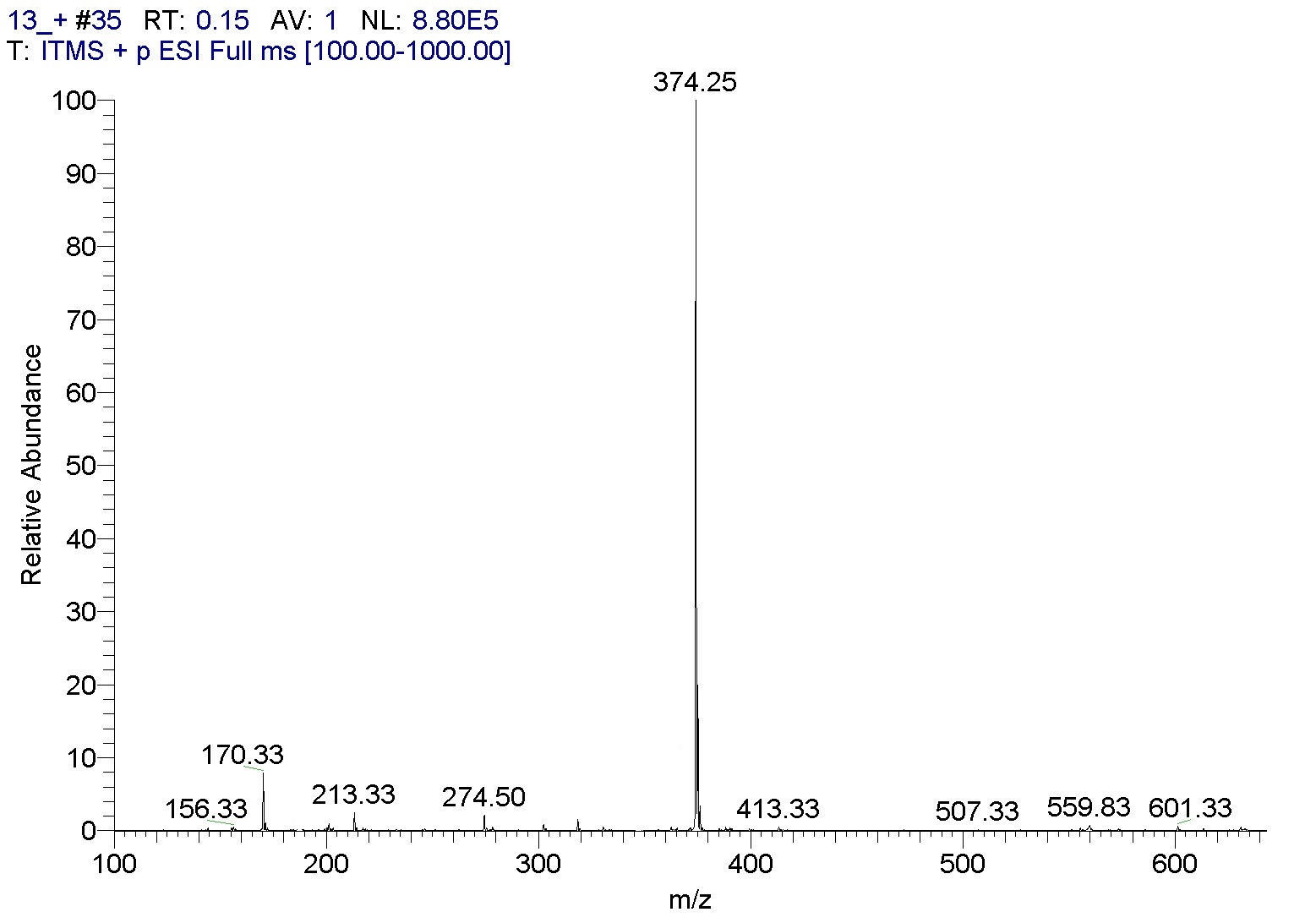
**

**
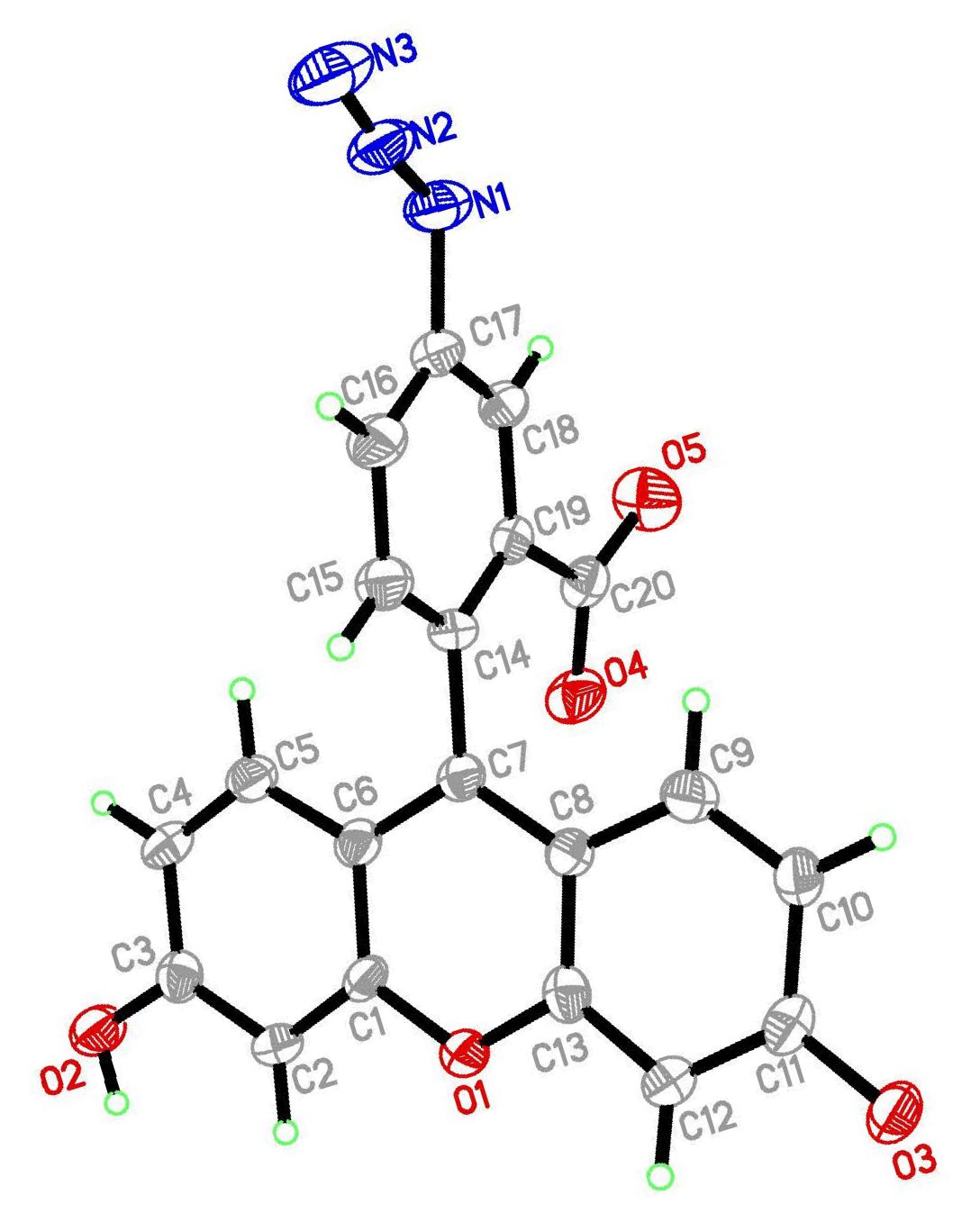
**

^1^H NMR (300 MHz, *d*_6_-DMSO): δ 6.52-6.60 (m, 4H, *J* = 22.9 Hz ), 6.66 (s, 2H), 7.28 (d, 1H, *J* = 8.1 Hz), 7.49 (s, 1H, *J* = 8.2 Hz), 7.62 (s, 1H), 10.12 (s, 2H); ^13^C NMR (75 MHz, *d*_6_-DMSO): δ 82.7, 101.7, 108.7, 112.1, 113.8, 125.1, 126.2, 127.4, 128.5, 141.2, 148.2, 151.3, 154.4, 129.0, 167.2, 168.6, 169.5. Elemental analysis (calcd. %) for C_20_H_11_N_3_O_5_: C, 64.17; H, 3.23; N 11.23, Found: C, 64.20; H, 3.20; N 11.22. ESI-MS m/z: [probe + H]^+^ Calcd for C_20_H_12_N_3_O_5_ 374.08 Found 374.25; Crystal data for C_20_H_12_N_3_O_5_: crystal size: 0.26 × 0.13 × 0.05, Monoclinic, space group P 1 21/n 1, *a* = 8.048(3) Å, *b* = 12.812(5) Å, *c* = 16.344(7) Å, *β* = 103.787(6), *V* = 1636.7(12) Å^3^, *Z* = 4, T = 173 K, *θ*_max_ = 27.45°, 10930 reflections measured, 3719 unique (*R*_int_ = 0.0663). Final residual for 255 parameters and 3719 reflections with *I*>2*σ* (*I*): *R*_1_ = 0.0944, *wR*_2_ = 0.1912 and GOF = 1.222.

**Figure S2:** Plot of the fluorescence intensity (at 531 nm) as a function of the concentrations of H_2_S

**
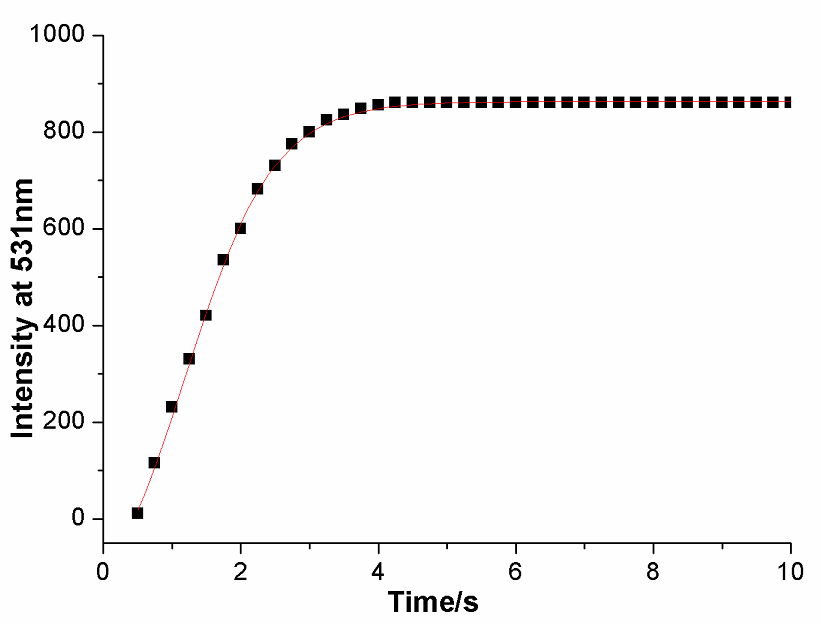
**

**Figure S3:** ESI-MS spectra of probe-H_2_S adduct

**
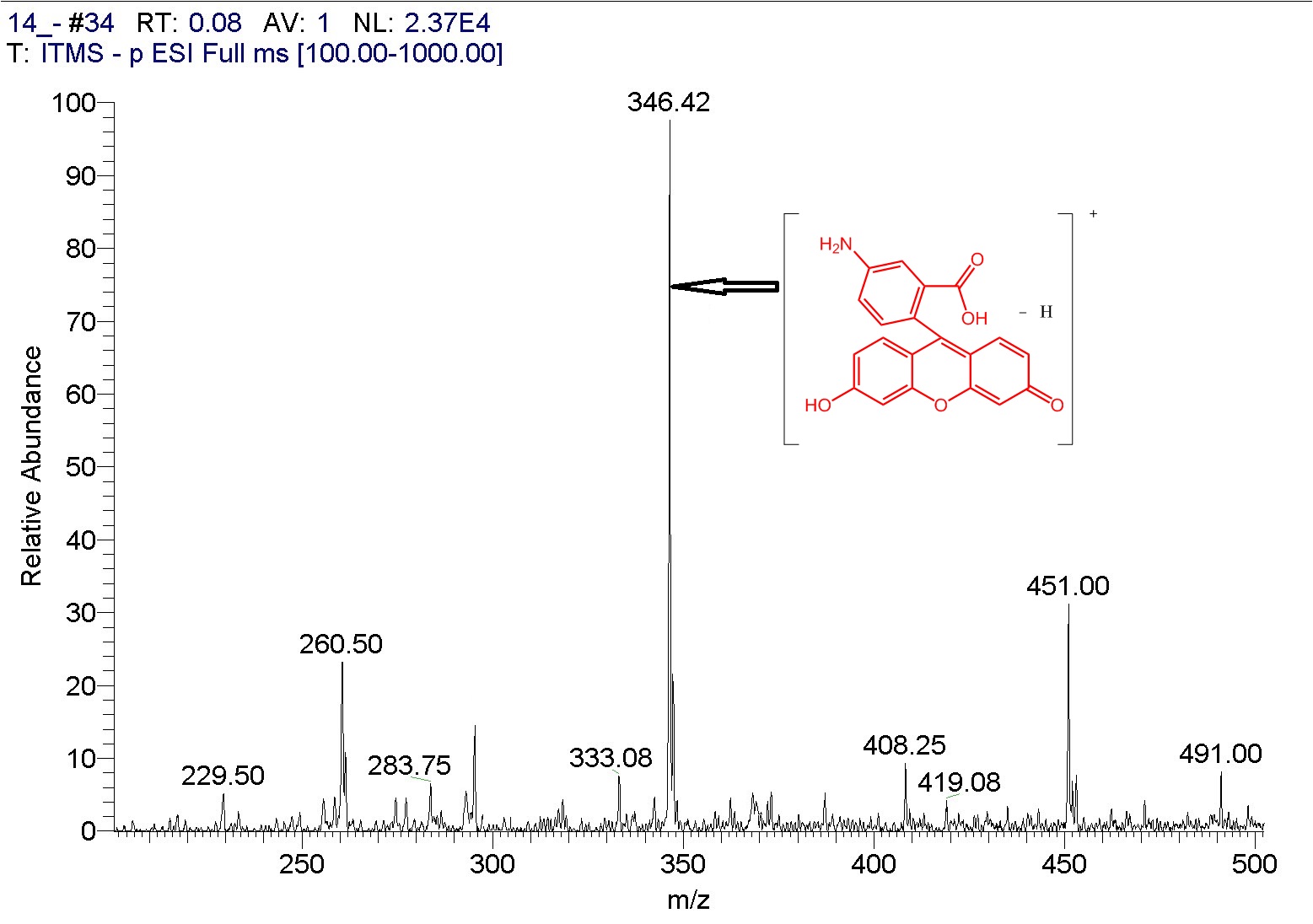
**

**Figure S4:** ^1^H NMR spectra of the product after probe reacting with H_2_S

**
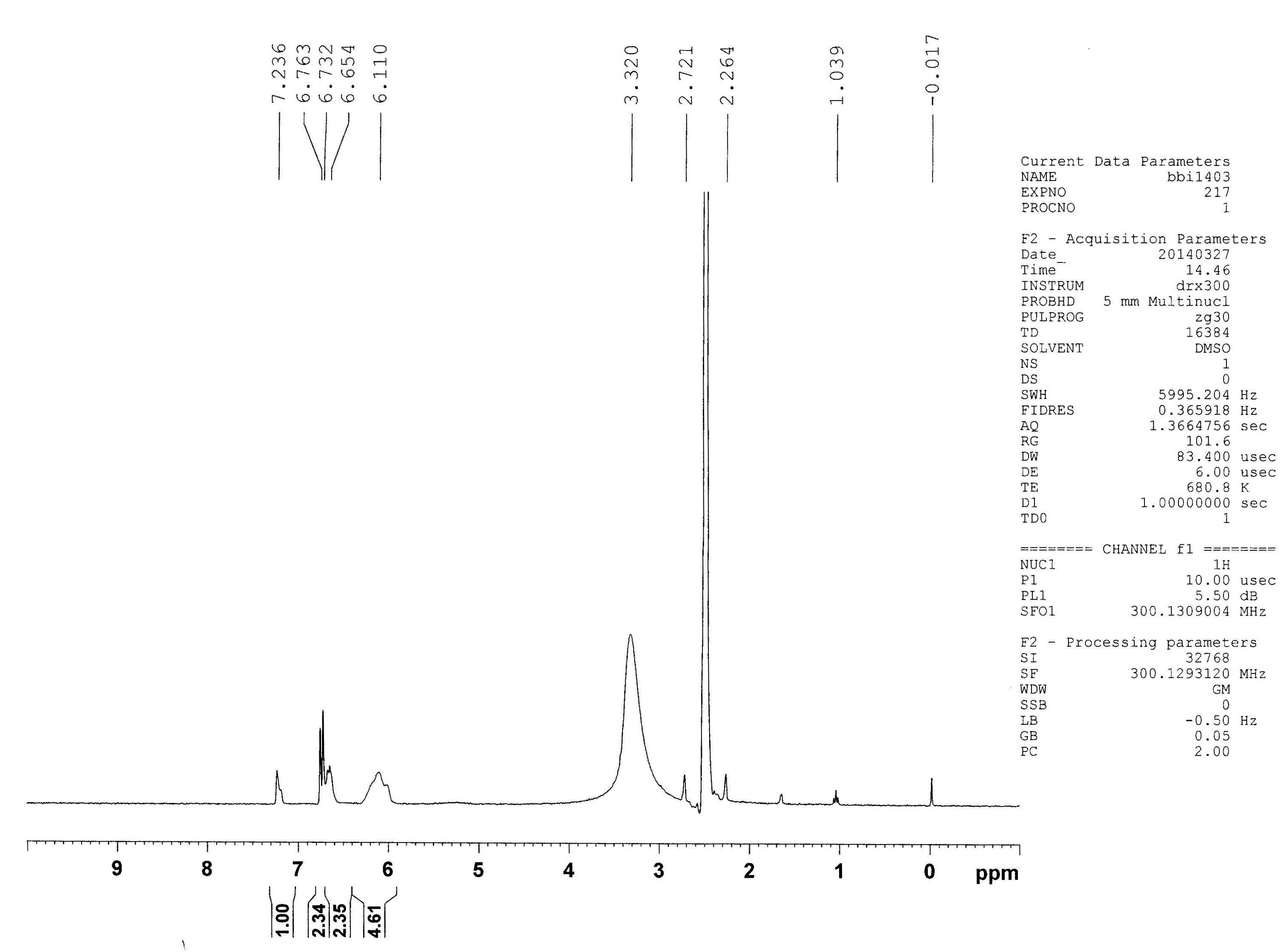
**

**Figure S5:** Detection limit for H_2_S from probe

**
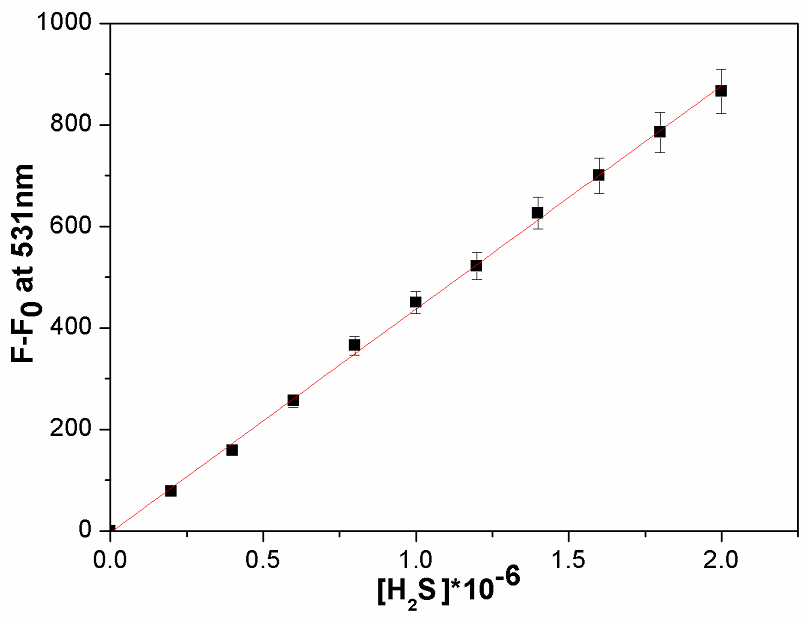
**

**Figure S6:** UV-Vis absorption spectra upon H_2_S addition into probe


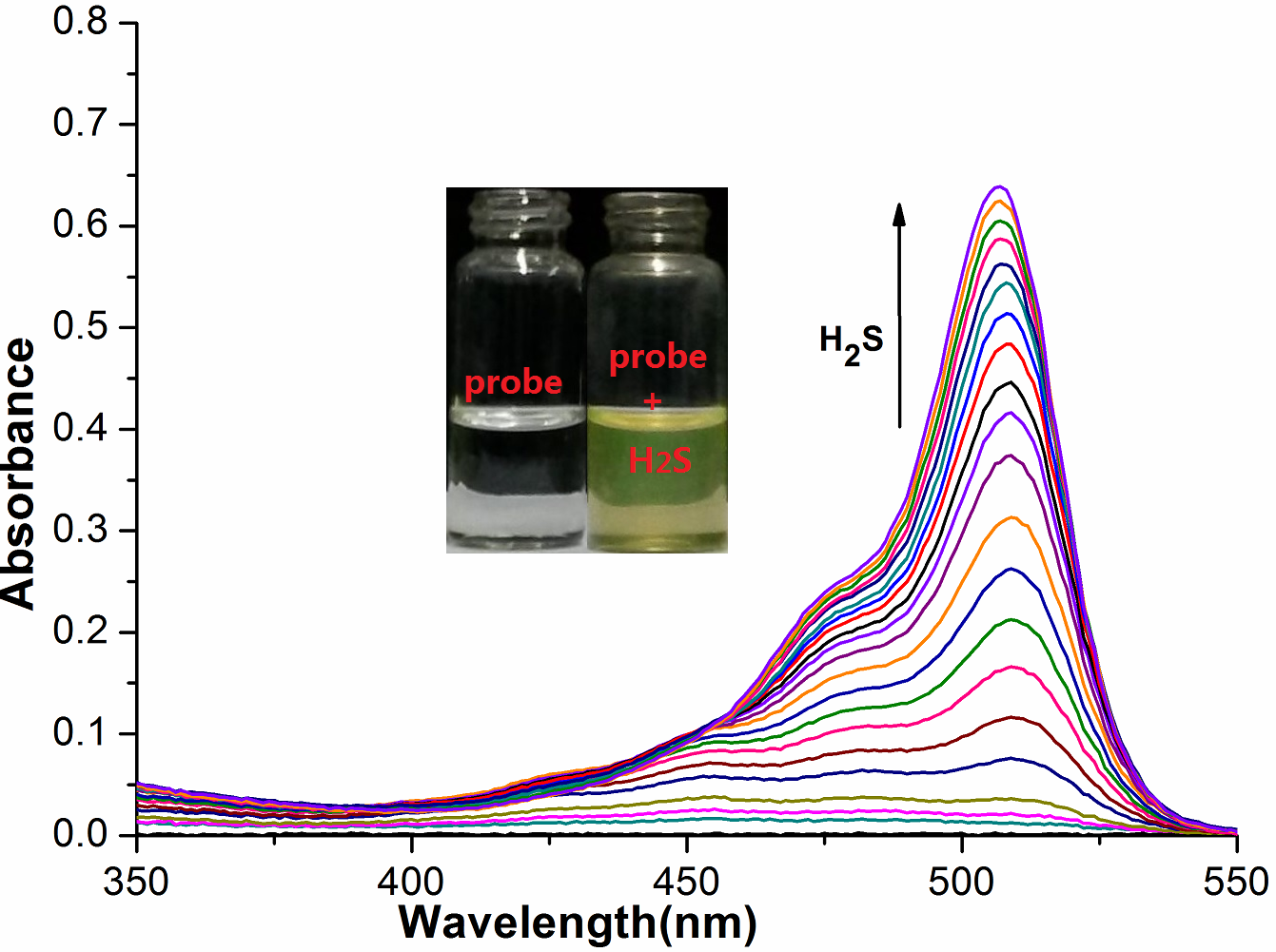

Supplement: Supplementary Information [file srep08969-s1.doc]
